# Survival outcomes after stereotactic body radiotherapy for 79 Japanese patients with hepatocellular carcinoma

**DOI:** 10.1093/jrr/rru130

**Published:** 2015-02-16

**Authors:** Hideomi Yamashita, Hiroshi Onishi, Naoya Murakami, Yasuo Matsumoto, Yukinori Matsuo, Takuma Nomiya, Keiichi Nakagawa

**Affiliations:** 1 Department of Radiology, University of Tokyo Hospital, 7-3-1, Hongo, Bunkyo-ku, Tokyo, 113-8655 Japan; 2 Department of Radiology, Yamanashi University; 3 Department of Radiology, National Cancer Center Hospital; 4 Department of Radiology, Niigata University School of Medicine; 5 Department of Radiation Oncology and Image-applied Therapy, Graduate School of Medicine, Kyoto University, Kyoto, Japan; 6 Department of Radiology, Yamagata University Hospital

**Keywords:** hepatocellular carcinoma, stereotactic body radiotherapy, SBRT

## Abstract

Stereotactic body radiotherapy (SBRT) is a relatively new treatment for liver tumor. Outcomes of SBRT for liver tumors unsuitable for ablation or surgical resection were evaluated.

A total of 79 patients treated with SBRT for primary hepatocellular carcinoma (HCC) between 2004 and 2012 in six Japanese institutions were studied retrospectively. Patients treated with SBRT preceded by trans-arterial chemoembolization were eligible. Their median age was 73 years, 76% were males, and their Child–Pugh scores were Grades A (85%) and B (11%) before SBRT. The median biologically effective dose (α/β = 10 Gy) was 96.3 Gy.

The median follow-up time was 21.0 months for surviving patients. The 2-year overall survival (OS), progression-free survival (PFS), and distant metastasis-free survival were 53%, 40% and 76%, respectively. Sex and serum PIVKA-II values were significant predictive factors for OS. Hypovascular or hypervascular types of HCC, sex and clinical stage were significant predictive factors for PFS. The 2-year PFS was 66% in Stage I vs 18% in Stages II–III. Multivariate analysis indicated that clinical stage was the only significant predictive factor for PFS. No Grade 3 laboratory toxicities in the acute, sub-acute, and chronic phases were observed.

PFS after SBRT for liver tumor was satisfactory, especially for Stage I HCC, even though these patients were unsuitable for resection and ablation. SBRT is safe and might be an alternative to resection and ablation.

## INTRODUCTION

In Japan, the infection rate of hepatitis C is high, with many cases of hepatocellular carcinoma (HCC) [1]. According to clinical practice guidelines from Japan, resection, radiofrequency ablation (RFA), and liver transplantation are the curative options available for HCC [2]. Recently, stereotactic body radiotherapy (SBRT) has become a treatment option for patients with liver tumors who are not eligible for surgery, RFA, or liver transplantation [3]. HCC has good radiation sensitivity [4]. However, currently SBRT of the liver is not frequently performed. This is because radiotherapy (RT) for liver tumors has been limited due to the risk of radiation-induced liver disease (RILD) [5]. However, technological advances have made it possible for radiation to be delivered to small liver tumors, while reducing the risk of RILD [6]. Resection, RFA, or trans-catheter arterial chemoembolization (TACE) are often performed for HCC in Japan. However, only 10–20% of HCC patients have resectable disease [7]. A drawback for RFA is that the procedure is difficult to perform in some anatomic areas [8]. Patients who are introduced to SBRT consist only of those with a central lesion of the liver, with direct invasion into the vessels, and/or with an insufficient outcome from TACE. In patients with centrally located HCC with chronic hepatitis or cirrhosis, major resection is often contraindicated due to insufficient residual liver volume [9]. RFA is therefore often contraindicated for HCC in those areas that are located in and near the hepatic portal vein or the central bile duct [10] and abutting the diaphragm [8]. Additionally, the risk of neoplastic seeding along the needle track after RFA has been reported [11].

The purpose of this study was to retrospectively evaluate the outcomes, mainly concerning survival, for patients treated at various dose levels in several Japanese institutions, although the local control rate has been reported elsewhere [12]. Because of the small number of cases of liver SBRT performed in each institution, it was necessary to gather results and data on side effects from many institutions in order to obtain meaningful information.

## MATERIALS AND METHODS

### Patients

This retrospective study reviewed data extracted from the database of the Japanese Radiological Society multi-institutional SBRT study group (JRS-SBRTSG) for 79 patients with HCC treated at six institutions (27, 19, 14, 9, 5 and 5 cases). The investigation period was from May 2004 to November 2012.

The diagnosis of HCC depended primarily on imaging studies, because pathological confirmation was not feasible in the candidates for SBRT. During follow-up of patients with liver disease, nodules ≥1 cm were diagnosed as HCC based on the typical hallmarks. These included being hypervascular in the arterial phase, with washout in the portal, venous or delayed phases about hypervascular HCC and, on the other hand, less-than-subtle density area in delayed phases and showing enlargement, plethoric change, and/or MRI signal change during long-time follow-up about hypovascular HCC from imaging studies. The imaging techniques included a combination of contrast-enhanced ultrasonography, 4-phase multi-detector computed tomography (CT), dynamic contrast-enhanced magnetic resonance imaging (MRI), and CT during hepatic arteriography and arterio-portography studies. The diagnosis was established according to a review of the imaging studies [13] and clinical practice guidelines [14–15]. The eligibility for SBRT for HCC was a single lesion. The version of staging classification used in this paper was the UICC classification version 7.

Patient and tumor characteristics are shown in Table 1. With regard to Child–Pugh scores before liver SBRT, 84.8% of patients had Grade A, 11.4% had Grade B, and 1.3% had Grade C. Hypovascular HCC was found in 16/79 cases (20%) and hypervascular HCC was found in 55/79 cases (70%). The feature of vascularity for the remaining eight patients was not evaluable in five patients, was unclear in one patient, and was not detectable by CT in two patients. The median alpha-fetoprotein (AFP) (ng/ml) and des-gamma carboxy prothrombin (PIVKA-II) (AU/ml) values before liver SBRT for 73 evaluable patients were 12.7 ng/ml (range, 0.8–8004) and 35 AU/ml (range, 3.1–16 900), respectively. The median indocyanine green retention rate at 15 min (ICG15) before liver SBRT for 25 evaluable patients was 21.2% (range; 3.0–56.2%). Liver SBRT was the first treatment in 26/79 cases (33%) and was also the first treatment for ectopic recurrences of liver SBRT in an additional seven cases.

**Table 1.** Patient and tumor characteristics of SBRT

Factors	*n*	Rate
All patients	79	100%
Stage		
I	29	37%
II	21	27%
III	7	9%
Recurrence	11	14%
NE	11	14%
Chilid–Pugh before SBRT		
A	67	85%
B	9	11%
C	1	1%
NE	2	3%
Sex		
Female	19	24%
Male	60	76%
Tumor maximum diameter (mm)		
Range	6–70	
Median	27	
Performance status (ECOG)		
0	34	43%
1	39	49%
2	4	5%
3	1	1%
Age (years old)		
Range	38–95	
Median	73	
SRT total dose (Gy)		
Range	40–60	
Median	48	
BED-10 (Gy)		
Range	75–106	
Median	96.3	

### Treatment

For treatment planning, abdominal pressure corsets such as a body shell (19 cases) and vacuum cushion (59 cases) such as blue back (5 cases), Vac-Lok (13 cases), or Body-Fix (5 cases) were used. In one case, none was used. Tumor motion was confirmed at < 1 cm in the cases using abdominal pressure. The gross tumor volume was delineated on both inspiratory and expiratory planning CT images by the respiratory depression method. The breath-holding method was used in 43 cases, the gating method in 10 cases, and the respiratory depression method in 25 cases. One patient was treated with free-breathing. The planning target volume was configured considering respiratory movement, the set-up margin, and the sub-clinical margin. SBRT was performed with an X-ray beam linear accelerator of 6 MV. The total irradiation dose delivered was dependent on the judgment rendered at each institution. A collapsed cone convolution, superposition algorithm, or analytical anisotropic algorithm was used for dose calculations.

The mode value of the total irradiation dose was 48 Gy in 4 fractions (38/79 cases) (from 40 Gy in 4 fractions to 60 Gy in 10 fractions). The prescription point was D95 (dose covering 95% volume within the PTV) in 48 patients (61%) and the iso-center in 31 patients (39%). The biologically effective dose (BED) (α/β = 10 Gy) was 75–106 Gy (median: 96 Gy) (Table 1). The following formula for BED_10_ was used: BED (Gy_10_) = nd × (1 + d/10). In all cases, CT registration such as kV cone beam CT or on-rail CT was performed during each treatment.

SBRT was delivered using multiple non-coplanar static beams (using >7 non-coplanar beams) generated by a linear accelerator or volumetric-modulated arc therapy. Daily image guidance, by using either orthogonal X-rays or onboard CT imaging, was used to re-localize the target before treatment delivery.

In seven patients, TACE was performed before SBRT. Oral tegafur/CDHP/oteracil potassium (S-1) was combined concurrently with liver SBRT in one patient.

### Follow-up

Patients were examined every 1 to 3 months for 1 year after liver SBRT and tri-monthly thereafter. Laboratory tests were performed at every visit. Treatment responses and intrahepatic recurrences were evaluated with dynamic contrast-enhanced CT or MRI every 3 months according to the modified Response Evaluation Criteria in Solid Tumors (mRECIST) [16]. Toxicity was evaluated with the Common Terminology Criteria for Adverse Events (CTCAE), version 4.0. Acute and sub-acute toxicities were deﬁned as adverse events occurring within 3 months and 3 to 6 months, respectively, after liver SBRT. Late toxicities related to liver and other toxicities were deﬁned as those occurring after 6 to 12 months and from 6 months to the last follow-up, respectively. Laboratory tests included determinations of aspartate aminotransferase, total bilirubin, platelet count, and albumin.

### Statistical analysis

Survival rates were calculated by Kaplan–Meier analysis. Log-rank testing was used to compare outcomes between the subsets of patients analyzed. Cox proportional hazards regression analysis was used for multivariate analysis. A *P*-value of < 0.05 was considered signiﬁcant. Data were analyzed with SPSS Statistics 20.0 (IBM Corp., Armonk, NY, USA). The points on survival curves by Kaplan–Meier were censored cases.

## RESULTS

### Eligible patients

The median follow-up time was 21.0 months (range, 3.4–68.3 months) for surviving patients. SBRT was performed as scheduled and was feasible in all patients. At the last follow-up, 48 cases (61%) had survived and 31 cases (39%) were deceased.

### Treatment outcomes

The first local effect was complete response in 36 cases (46%), partial response in 28 cases (35%), no change in 9 cases (11%), progressive disease in 4 cases (5%), and not evaluable in 2 cases (3%). At censoring during the follow-up, 14 cases (18%) had local progression, 63 cases (80%) did not have local progression, and 2 cases (3%) were not evaluable.

For the 79 patients, the 2-year overall survival (OS), progression-free survival (PFS), and distant metastatic-free survival (DMF) were 52.9% ± 7.1%, 39.9% ± 6.9%, and 76.3% ± 6.6%, respectively. The number of patients at risk was 43 (54%), 21 (27%), 9 (11%), and 3 (4%) at 1-, 2-, 3- and 4-years in OS, respectively.

**Fig. 1.**
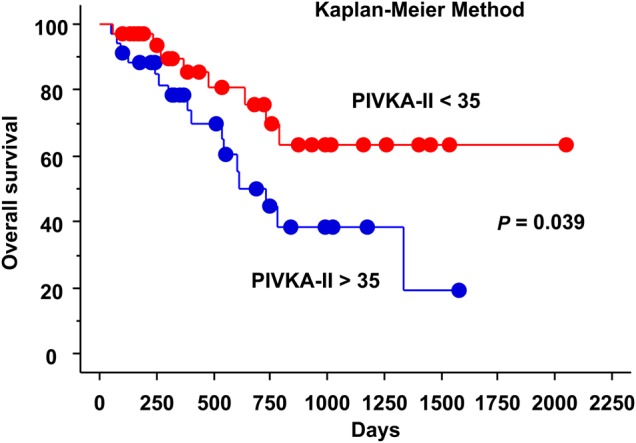
Overall survival curves by serum PIVKA-II value (over 35 vs under 35 AU/ml). There was no patient with serum PIVKA-II level of just 35 AU/ml.

**Fig. 2.**
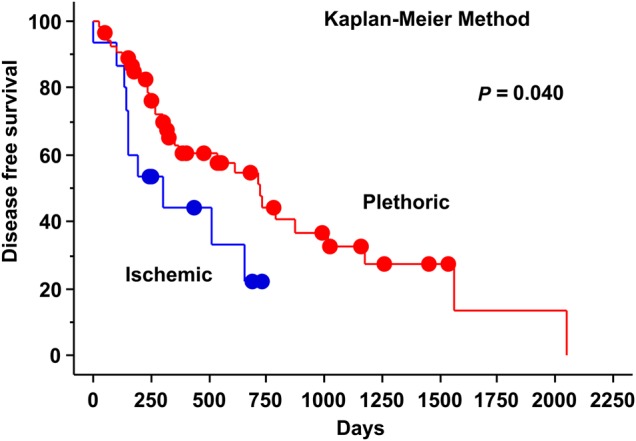
Progression-free survival curves by HCC type (hypovascular vs hypervascular).

**Fig. 3.**
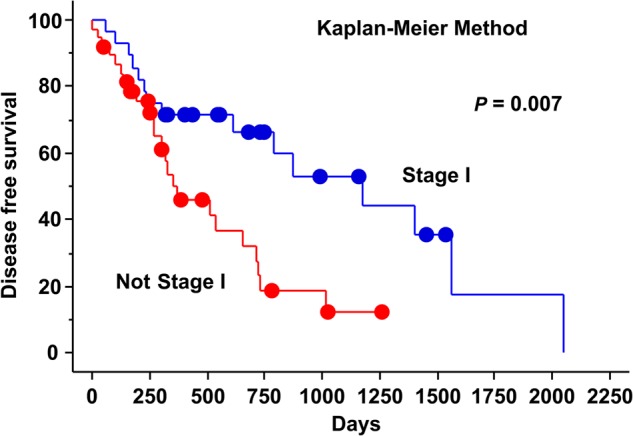
Progression-free survival curves by clinical stage (I vs II–III).

The results of sub-analysis of survival are shown in Table 2. Sex (female vs male) and serum PIVKA-II value (over 35 vs under 35) (Fig. 1) were significant predictive factors for 2-year OS (*P* = 0.044 and 0.039, respectively) by the log-rank test. HCC type (hypovascular vs hypervascular) (Fig. 2), sex (female vs male), and clinical stage (I vs II–III) (Fig. 3) were significant predictive factors for 2-year PFS (*P* = 0.040, 0.049 and 0.007, respectively) by the log-rank test.

**Table 2.** Subanalysis of survival

Factors	2-year OS	*P-*value by log-rank	2-year PFS	*P-*value by log-rank
Chemotherapy				
With	47.6 ± 18.7	0.10	41.4 ± 7.1	0.75
Without	53.6 ± 7.6		24.1 ± 19.5	
Tumor diameter				
Over 30 mm	55.9 ± 10.2	0.70	35.0 ± 9.2	0.34
Under 30 mm	50.3 ± 10.7		50.3 ± 9.8	
HCC type				
Hypovascular	43.2 ± 20.8	0.86	22.2 ± 13.0	0.040
Hypervascular	51.6 ± 8.4		44.2 ± 8.2	
Child–Pugh Grade				
A	53.6 ± 8.0	0.13	40.5 ± 7.5	0.22
B–C	30.3 ± 17.1		36.0 ± 16.1	
Sex				
Female	78.4 ± 11.2	0.044	67.6 ± 12.1	0.049
Male	43.1 ± 8.4		30.3 ± 7.7	
Serum AFP value				
Over 20	52.3 ± 10.9	0.81	42.1 ± 10.2	0.59
Under 20	54.8 ± 9.9		45.3 ± 9.6	
Serum PIVKA-II				
Over 35	44.7 ± 10.6	0.039	32.5 ± 9.8	0.16
Under 35	69.7 ± 9.9		54.4 ± 9.8	
BED (Gy)				
Over 100	48.1 ± 10.4	0.28	41.8 ± 10.2	0.99
Under 100	57.2 ± 9.7		39.2 ± 8.8	
Age (years old)				
Over 75	56.7 ± 10.2	0.80	54.4 ± 9.3	0.58
Under 75	49.7 ± 9.8		30.2 ± 8.6	
Hilum LN metastasis				
With	50.0 ± 35.4	0.32	0 ± 0	0.12
Without	53.5 ± 7.3		41.9 ± 6.9	
Clinical stage				
I	58.2 ± 10.8	0.40	66.3 ± 9.3	0.007
II-	50.0 ± 10.9		18.4 ± 8.0	
Primary effect				
PR/CR	56.8 ± 7.8	0.44	42.9 ± 7.5	0.24
NC/PD	38.7 ± 19.5		28.7 ± 15.3	
Performance status				
0–1	54.5 ± 7.6	0.15	37.6 ± 6.9	0.26
2-	50.0 ± 25.0		50.0 ± 25.0	

OS = overall survival, PFS = progression free survival, HCC = hepatic cell carcinoma, AFP = α-fetoprotein, PIVKA = protein induced by vitamin K absence or antagonist, PR = partial response, CR = complete response, NC = no change, PD = progressive disease.

By multivariate analysis (Cox proportional hazards regression analysis), clinical Stage I vs II–III (other covariates were male vs female and PIVKA-II > 35 vs < 35) was the only significant predictive factor for PFS (*P* = 0.017, 95% CI = 0.190–0.848) (Table 3). No differences in predictive factors were shown for OS and PFS, even when other factors such as tumor diameter ≥30 mm vs <30 mm, hypervascular vs hypovascular HCC by CT scan, and BED_10_ ≥100 Gy vs <100 Gy were added to the analysis.

**Table 3.** Multivariate analysis for survival

Factors	OS		PFS	
	*P-*value	95% CI	*P-*value	95% CI
Stage	0.47		0.017	
I		0.303–1.730		0.190–0.848
II–		1		1
Sex	0.29		0.36	
Male		1		1
Female		0.123–1.871		0.246–1.665
PIVKA-II	0.28		0.56	
Over 35		0.656–4.330		0.604–2.547
Under 35		1		1

OS = overall survival, PFS = progression free survival.

### Treatment-related toxicity

All liver SBRTs were completed without toxicity during the RT period. There was no Grade 5 toxicity. After the RT period, six patients (4.6%) experienced Grade 3–4 gastrointestinal toxicity and three patients (2.3%) had Grade 2 gastrointestinal toxicity. With regard to Grade 3–4 toxicities, duodenal ulcer, transverse colon ulcer, gastroduodenal aorta rupture, biliary stricture after SBRT occurred in one patient, respectively, and gastrointestinal bleeding in two patients. Only the gastroduodenal aorta rupture was Grade 4 toxicity. Of these nine patients, seven had a Child–Pugh score of Grade A, and the other two patients had a Child–Pugh score of Grade B before SBRT. No significant ( ≥ Grade 3) liver enzyme elevation was observed during treatment, nor was classic RILD observed.

## DISCUSSION

This is a retrospective study that reviewed data extracted from the database of JRS-SBRTSG for 79 patients with HCC treated at six institutions. The OS of 53% in this study at 2 years after liver SBRT might be considered satisfactory considering that the patient group included frail patients for whom surgery was contraindicated due to decompensated cirrhosis and who were in an older age group (median age 73 years). Patients in this study were very heterogeneous, and some patients might not have been candidates for SBRT according to strict guidelines. Survival data was the only factor analyzed in this study.

Survival data after SBRT for liver tumor from previous reports are summarized in Table 4. According to those reports, the 2-year OS was 34% [15], 52% [16], 55% [21], 60% [18], 67% [20] and 83% [19]. The 2-year OS was 53% in the present study, which cannot be viewed as a satisfactory result. In order to improve our results for survival, an increase in the radiation dose may be required, although BED_10_ was not the factor for survival in the present study (Table 2). The median BED_10_ in this study was 96 Gy; therefore, over half of the patients received a BED_10_ of <100 Gy. Dose escalation for HCC patients with decompensated cirrhotic liver disease may be deleterious with respect to normal liver tolerance. Takeda *et al.* [23] used 35–40 Gy in five fractions (59.5–72.0 Gy in BED_10_), based on baseline liver function and on liver volume receiving ≥20 Gy (V20) in SBRT for untreated solitary HCC patients. They reported relatively good results, in which the 2-year local control rate and OS were 95% and 87%, respectively [23], although the BED_10_ was not very high. In their paper [23], the doses were prescribed to the planning target volume surface. In the present study, on the other hand, the doses were prescribed to the PTV-D95 (61%) or the iso-center (39%).

**Table 4.** Previous reports on survival after SBRT for HCC

Year	Ref	Dose	Subject	*n*	MST (mo)	OS	PFS	Median size	Child
2008	[17]	Median 36 Gy/6 Fr	HCC	31	11.7	1 year: 48%		173 cm^3^	
2010	[18]	Median 36 Gy/3 Fr	HCC	17		1 year: 75%			
						2 year: 60%			
2010	[19]	30–39 Gy	HCC	42		1 year: 93%	1 year: 72%	15.4 cm^3^	
						3 year: 59%	3 year: 68%		
			HCC	25		1 year: 79%		4.5 cm	A: 48%
2010	[16]	45 Gy/3 Fr by Cyber				2 year: 52%			B: 4%
									C: 28%
2011	[20]	Median 44 Gy/3 Fr		60		2 year: 67%	2 year: 48%	3.2 cm	A: 60%
									B: 40%
2012	[21]	Median 30 Gy/15 Fr	HCC	21		1 year: 87%			
			ICC	11		2 year: 55%			
2013	[15]	Median 36 Gy/6 Fr	HCC	102	17.0	1 year: 55%		117 cm^3^	A: 100%
						2 year: 34%			
2013	[22]	Median 60 Gy	HCC	14	37.0	1 year: 83%			
						2 year: 83%	2 year: 54%	2.3 cm	

MST = median survival time, OS = overall survival, PFS = progression-free survival, Child = Child–Pugh Grade.

By multivariate analysis, clinical Stage I vs Stage II–III was the only significant prognostic factor for PFS. The main prognostic factors of HCC reported previously included stage classification, invasion to a blood vessel, liver function, tumor diameter, or the number of tumors [24–26]. However, in our study, clinical stage was found to be the sole prognostic factor.

Guidelines for HCC diagnosis indicate that a pathological diagnosis is not necessary if a tumor has a typical radiographic appearance. In this study, 20% of the patients had hypovascular HCC, and most of these HCC lesions were diagnosed by ^18^fluorine-fluorodeoxyglucose positron-emission tomography study and the α-fetoprotein tumor marker of the L3 fraction. The reason for the poorer survival of patients with the hypovascular type of HCC than patients with the hypervascular type was not clear. Usually, hypovascular HCC is at an earlier stage and has a good prognosis. This reason why hypovascular HCC had a poorer prognosis may be that many cases of hypovascular HCC in this study had been observed without immediate treatment until size-up, plethoric change, and/or MRI signal change, as stated above.

Only one patient with Child–Pugh Grade C was treated with SBRT in this study. In that patient, there was no other treatment option, and the patient was informed of the risks of the procedure and provided consent.

There are some limitations in this study in that it is retrospective and part of a multi-institutional series with a relatively short follow-up period (median 15 months). In addition, the irradiation dose and follow-up methods were inconsistent. The reason for the lack of difference according to the stratification of the irradiation dose may be due to the various algorithms or to the differing prescription points between institutions.

## CONCLUSION

Overall survival after SBRT for liver tumor was satisfactory, especially in Stage I HCC, despite the candidates being unsuitable for resection and ablation. SBRT is safe and might be an alternative method to resection and ablation.

## FUNDING

Funding to pay the Open Access publication charges for this article was provided by KAKENHI of Grants-in-Aid for Scientific Research.
